# Alteration of Fractional Anisotropy and Mean Diffusivity in Glaucoma: Novel Results of a Meta-Analysis of Diffusion Tensor Imaging Studies

**DOI:** 10.1371/journal.pone.0097445

**Published:** 2014-05-14

**Authors:** Ke Li, Cuixin Lu, Yufei Huang, Li Yuan, Dong Zeng, Kan Wu

**Affiliations:** 1 Key Laboratory for NeuroInformation of Ministry of Education, University of Electronic Science and Technology of China, Chengdu, China; 2 School of Life Science and Technology, University of Electronic Science and Technology of China, Chengdu, China; University of Electronic Science and Technology of China, China

## Abstract

**Objectives:**

We hypothesized that a meta-analysis of existing studies may help to reveal significant changes on diffusion tensor imaging (DTI) in patients with glaucoma. Therefore, a meta-analysis was utilized to investigate the possibility that DTI can detect white matter damage in patients with glaucoma.

**Methods:**

The study design and report adhered to the PRISMA Statement guidelines. DTI studies that compared glaucoma patients and controls were surveyed using PubMed, Web of Science and EMBASE (January 2008 to September 2013). Stata was used to analyze the decrease in fractional anisotropy (FA) and increase in mean diffusivity (MD) in the optic nerve and optic radiation in patients with glaucoma.

**Results:**

Eleven DTI studies were identified through a comprehensive literature search, and 10 independent DTI studies of glaucoma patients were eligible for the meta-analysis. A random effects model revealed a significant FA reduction in the optic nerve and optic radiation, as well as a significant MD increase in the tracts. A heterogeneity analysis suggested that FA may be related to glaucoma severity.

**Conclusions:**

Our findings revealed that the optic nerve and optic radiation were vulnerable regions in patients with glaucoma and that FA may be correlated with glaucoma severity and age. Furthermore, this study suggests that magnetic resonance imaging in patients with glaucoma may help to provide objective evidence to aid in the diagnosis and management of glaucoma.

## Introduction

Glaucoma is the second most common cause of blindness in the world [1], and it currently affects approximately 90 million people worldwide [2], [3]. However, the primary cause and pathogenesis of this disorder remain unclear [4]. Recently, glaucoma has been found to be a neurodegenerative disease that is only partially influenced by ocular factors [5], [6]–[8]. Glaucoma is believed to be an optic neuropathy characterized by loss of retinal ganglion cells correlated with visual field defects and alterations of the optic nerve [3]. Moreover, neuronal degeneration involving all parts of the central visual pathways has been documented at autopsy, and severe visual field losses have been observed in both eyes [9]. Diffusion tensor imaging (DTI) can quantitatively measure anterior visual pathway compression using fractional anisotropy (FA) and mean diffusivity (MD) [10]. An FA decrease and MD increase may indicate structural damage to the optic nerve axon in patients with glaucoma [11].

Abnormalities of the optic nerves, optic chiasm, optic tracts, and optic radiations have been repeatedly investigated in DTI studies of patients with glaucoma [12], [13]. Similarly, structural changes have been detected in the right and left inferior occipital gyri, right middle occipital gyrus, right inferior temporal gyrus, and right occipital lobe [14]. In addition, DTI studies have demonstrated decreased FA in the optic nerves and optic radiation [13].

The parameter FA measures the orientation coherence of diffusion and provides information regarding fiber integrity [15], [16]. Meanwhile, FA has a negative correlation with visual parameters in the optic nerves [17]. Additionally, several quantitative studies indicate that FA may be correlated with glaucoma severity [18], [19]. Similarly, the height of the optic chiasm is correlated with the MD value [20], and visual field defects have been associated with changes in the DTI parameters of the optic nerve, such as an increased MD [6], [21].

Although several DTI studies in patients with glaucoma have investigated brain damage, they have yielded inconsistent results. It has been reported that the MD is significantly increased in the right optic radiation and in the posterior region of the left optic radiation in glaucoma patients [19], [22]–[24], while other studies have reported no significant MD changes [25]. In addition, some studies indicate that the MD of the optic nerves is correlated with glaucoma severity [26], [27]. However, other studies suggest that the MD increase in the optic nerve is not correlated with the stage of glaucoma [18]. Outcomes have been inconsistent, possibly due to small sample sizes.

Thus, a meta-analysis of the existing studies may help to demonstrate significant changes in DTI results in patients with glaucoma by utilizing a larger sample size and enabling greater power in the statistical analysis. We hypothesized the following: that the microstructural brain damage that occurs in patients with glaucoma can be detected with DTI, that FA is reduced in the optic nerves and optic radiation in glaucoma patients, that MD is increased in the optic tracts, and that MD is not correlated with glaucoma severity. To test these hypotheses and verify whether DTI can serve as a biomarker of glaucoma, we performed a literature search of DTI studies in glaucoma patients and conducted a meta-analysis with the data from these studies.

## Materials and Methods

### Data Sources

The study design and report adhered to the PRISMA Statement guidelines (supporting information Figure S1 and Checklist S1).DTI studies that examined the FA and MD in glaucoma patients, compared with those in controls, were obtained through an extensive search of three databases: PubMed, Web of Science, and EMBASE. They were searched using the following terms: ‘glaucoma’, ‘DTI’, ‘Diffusion tensor imaging’, ‘Diffusion tensor Magnetic Resonance Imaging’, and ‘DTMRI’. To identify relevant studies, two reviewers performed independent screenings of the titles and abstracts of the studies. In those studies, related parameters and information regarding brain changes were included.

### Selection Criteria

**Figure 1:**
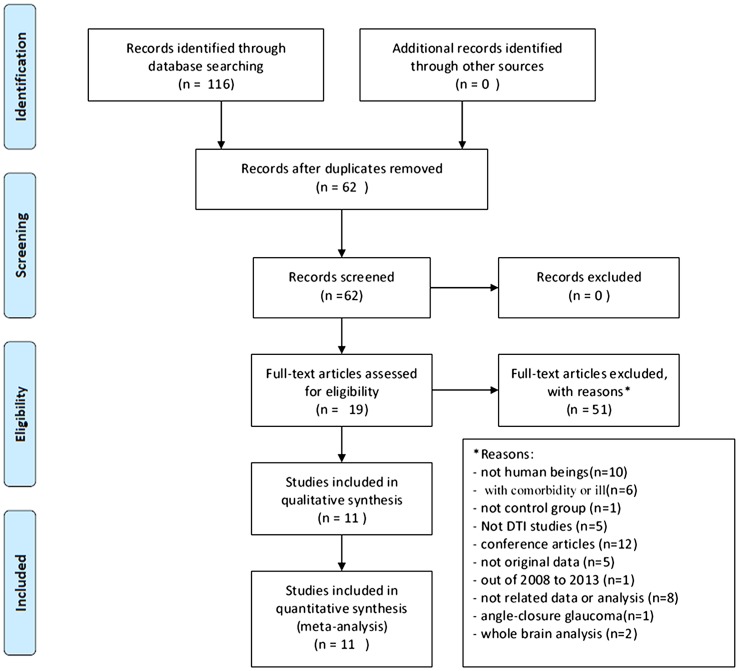
Process of study selection.

To choose the most detailed and most readily available studies, the inclusion criteria for the articles were as follows: (1) the data were acquired from adult glaucoma patients in the original studies; (2) the studies reported the FA and MD values of the glaucoma and control groups; (3) articles were limited to those published in peer-reviewed, English-language journals between January 2008 and September 2013; (4) the studies included samples without angle-closure glaucoma; (5) the studies reported sufficient data (FA and MD values in the optic nerve and optical radiation) to enable effective calculations; and (6) the analysis method was a region of interest analysis. The process of study selection is shown in Figure 1.

### Data Extraction

To ensure that the meta-analysis was adequately powered, we emailed the corresponding author to obtain more information if a study did not report sufficient data. If the author did not respond, we excluded the study from our analysis.

Eleven demographic, methodological, and clinical variables, including the number of participants, number of male participants, mean age, strength of the magnetic field (Tesla), analysis method, acquisition matrix, echo time (TE), repetition time, field of view, b-value, and thickness, were extracted and are shown in Table 1.

**Table 1:** Data used in the meta-analysis.

Studies	No. glaucoma	Mean Age	Magnetic field intensity	Analysis method	Acquisition matrix	Echo time	Repetition time	Field of View	b-value	Slice Thickness
Chen-2013	25	34.5	3	ROI	128*128	13000	69	240*240	800	2
Dai-2013	25	34.5	3	ROI	128*128	13000	68.5	256*256	800	2
Murai-2013	29	59	3	ROI	256*256	4300	90	240*240	1000	5
Zhang-2012	30	54.8	1.5	ROI	128*128	9000	120	230*230	1000	4
Chang-2013	16	63	1.5	No mentioned	256*256	4805	59	250*250	800	2
Bolacchi-2012	24	59	3	ROI	256*256	41355	59	250*250	800	2
Engelhorn-2012	20	/	3	ROI	512*512	3400	93	230*230	1000	4
Michelson-2012	20	/	3	ROI	512*512	3400	93	230*230	1000	4
Wang-2013	23	/	3	ROI	256*256	5000	97	230*230	1000	2
Garaci-2009	16	63	3	ROI	256*256	11000	120	250*250	800	2
Simone-2013	13	63	3	ROI	256*256	3400	93	250*250	1000	5

### Glaucoma Classification

The glaucomatous eyes were stratified according to severity of visual field defects into three groups using the Hodapp–Anderson–Parrish system. Early glaucoma was characterized by a mean deviation score (MDS) of −0.01 to −6.00 dB; moderate glaucoma was characterized by an MDS of −6.01 to −12.00 dB; and severe glaucoma was characterized by an MDS of greater than −12 dB [13], [18].

### Statistical Analysis

All meta-analyses were performed using Stata12 (published by Stata Press, 4905 Lakeway Drive, College Station, Texas 77845). Stata statistical software is an integral, comprehensive statistical software package for data analysis, data management, and graphics. In addition, the datamanagement features of stataenable complete control of all types of data, and the functions enable the combining and reshaping of datasets, management of variables, and collection of statistics across groups or replicates. A synthetic series of customer-written commands in Stata is freely available for use in meta-analysis. We can analyze binary parameters associated with a meta-analysis (relative risk, risk difference, odds ratio) and successive outcomes (difference in means, standardized difference in means). Stata can draw forest plots, funnel plots, and L'Abbéplots and perform statistical tests for funnel plot asymmetry. Stata makes it simple to generate publication-quality and distinctly styled graphs(http://www.stata.com/features/).

The standardized mean difference (SMD) was calculated and used for the effect sizes. The SMD is the standardized difference between two means and can be calculated as the difference between the glaucoma and control groups divided by the pooled standard deviation (SD) [28]. Cohen's pooled SMD was used to characterize the change in FA or MD. We performed a statistical analysis of the variables that were reported in more than three studies. The SMD of the FA and MD in the optic nerve and optic radiation between the glaucoma and control groups was calculated as part of the statistical analysis.

### Subgroup Analyses

To control for possible methodological differences between the studies, repeated analysis was performed only on studies that were methodologically homogenous. Subgroup analysis was performed to verify the source of heterogeneity. Subgroups were divided by the extracted data items. FA may potentially be related to glaucoma severity, and mean age was a key factor affecting glaucoma severity [26], [29], [30].

Heterogeneity was assessed using I2 statistics and was calculated as the weighted sum of the squared differences between individual study effects and the pooled effect across studies [31].Thresholds for the interpretation of I2 were as follows: 0% to 50%, low heterogeneity; 50% to 75%, medium heterogeneity; and 75% to 100%, high heterogeneity.

## Results

### Included Studies

The results of the literature search and number of studies included and excluded are shown in Figure 1. In this meta-analysis, ten studies were included in the FA analysis of the optic nerve and optic radiation, involving 202 individuals with glaucoma and 218 controls. The clinical characteristics and demographics of the participants are shown in Table 2. Eight studies were included in the MD analysis of the optic tracts and optic radiation, involving 192 individuals with glaucoma and 167 controls. All eight studies included adult glaucoma MD samples (Table 3).

**Table 2:** Information statistics: on the mean and standard deviation of the FA in the optic nerve and optic radiation.

Optic nerve Study	Glaucoma patients				Controls		
	No.	FA Mean	FA SD	Stage of glaucoma	NO.	FA Mean	FA SD
Chen [22]	25	0.35	0.087	All stages	24	0.47	0.087
Dai [19]	25	0.16	0.020	All stages	25	0.20	0.020
Murai [48]	29	0.45	0.048	Mild and severe	19	0.51	0.051
Zhang [38]	30	0.39	0.090	All stages	30	0.54	0.080
Bolacchi-1 [10]	12	0.39	0.043	Early stage	24	0.52	0.050
Bolacchi-2 [10]	10	0.26	0.034	Severe stage	24	0.52	0.050
Engelhorn [40]	20	0.48	0.150	All stages	20	0.66	0.120
Michelson [49]	22	0.48	0.150	All stages	22	0.66	0.120
Wang [18]	23	0.26	0.089	Mild and severe	20	0.59	0.035
Garaci [13]	16	0.22	0.013	Severe stage	10	0.52	0.066

Optic radiation Study	Glaucoma patients				Controls		
	No.	FA Mean	FA SD	Stage of glaucoma	No.	FA Mean	FA SD
Chen [22]	25	0.58	0.050	All stages	24	0.64	0.050
Dai-1 [19]	25	0.40	0.030	All stages	25	0.46	0.030
Dai-2 [19]	25	0.38	0.040	All stages	25	0.44	0.040
Murai [48]	29	0.44	0.046	Mild and severe	19	0.51	0.050
Bolacchi-1 [10]	12	0.40	0.053	Early stage	24	0.56	0.060
Bolacchi-2 [10]	10	0.29	0.058	Severe stage	24	0.56	0.060
Engelhorn-1 [40]	20	0.40	0.160	Mild stage	20	0.53	0.200
Engelhorn-2 [40]	20	0.48	0.170	Early stage	20	0.64	0.211
Michelson [49]	22	0.50	0.200	All stages	22	0.66	0.110
Li [50]	13	0.45	0.020	Severe stage	7	0.47	0.020

FA: fractional anisotropy; SD: standard deviation.

**Table 3:** Mean diffusivity (mean and standard deviation) in the optic tract (top) and optic radiation (bottom).

optic tract Study	Glaucoma			Control	
	No.1	Mean and SD	Stage of glaucoma	No.2	Mean and SD
Bolacchi [10]	25	1.90±0.22×10−3	Severe stage	24	0.86±0.15×10^−^3
Chen [22]	24	1.61±0.25×10^−^3	All stages	24	1.40±0.25×10^−^3
Dai [19]	25	0.71±0.05×10^−^3	All stages	25	0.62±0.05×10^−^3
Zhang [38]	30	1.94±0.31×10−3	All stages	30	1.48±0.17×10^−^3
Chang [51]	27	1.33±0.31×10−3	All stages	12	0.91±0.31×10−3
Michelson [49]	22	0.74±0.21×10^−^3	All stages	22	0.58±0.17×10^−^3
Wang [18]	23	1.81±0.37×10^−^3	Mild and severe	20	1.03±0.12×10^−^3
Garaci [13]	16	1.61±0.20×10^−^3	Severe stage	10	1.06±0.16×10^−^3

optic radiation Study	Glaucoma			Control	
	No.1	Mean and SD	Stage of glaucoma	No.2	Mean and SD
Bolacchi [10]	25	1.08±0.18 ×10^−^3	Severe stage	24	0.90±0.27×10^−^3
Chen [22]	24	0.42±0.25×10^−^3	All stages	24	0.40±0.25×10^−^3
Dai [19]	25	0.71±0.05×10^−^3	All stages	25	0.65±0.05×10^−^3
Michelson [49]	22	0.70±0.16×10^−^3	All stages	22	0.55±0.16×10^−^3
Garaci [13]	16	0.94±0.06×10^−^3	Severe stage	10	0.78±0.031×10^−^3

SD: standard deviation.

### Meta-analysis of DTI Measures

**Figure 2:**
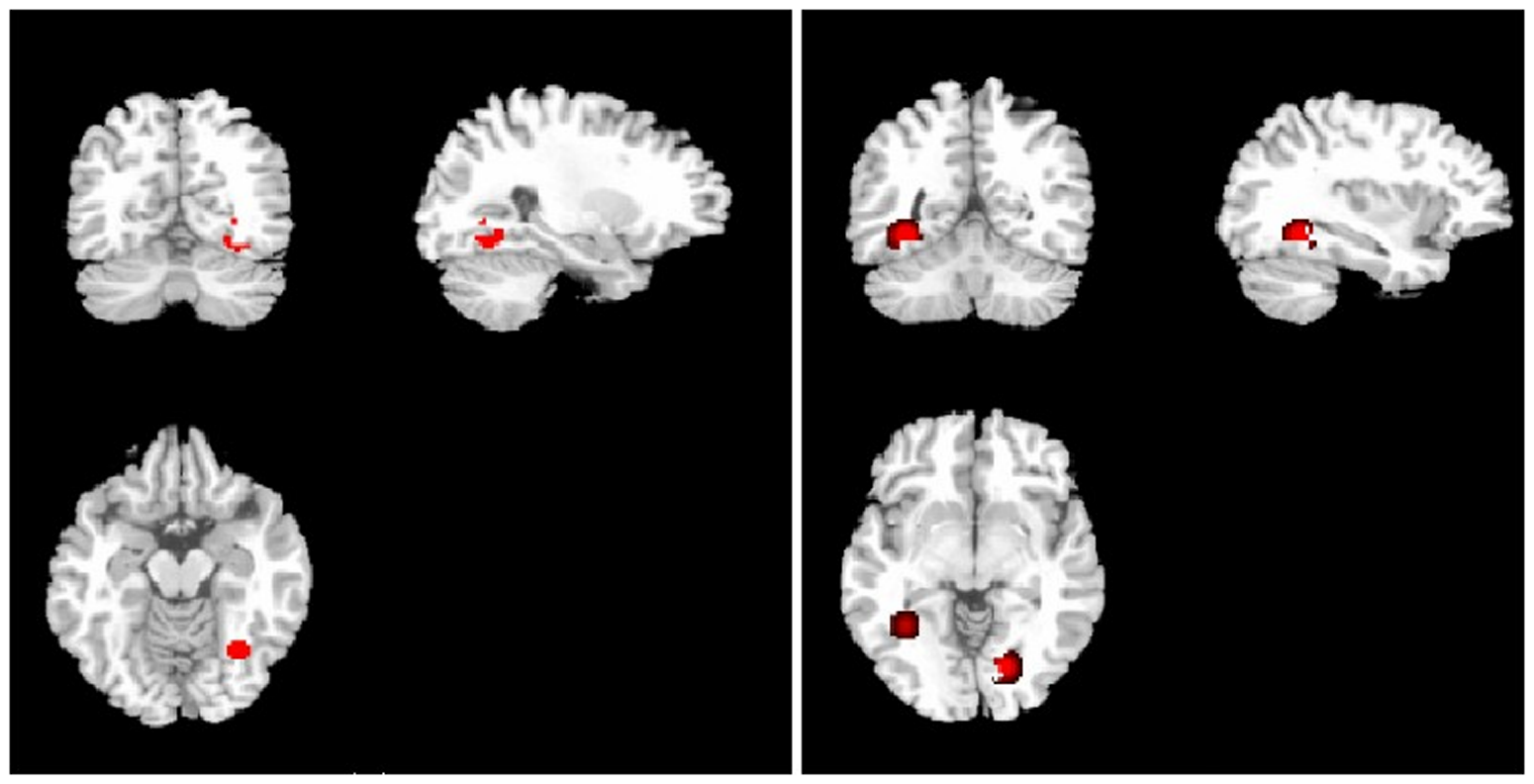
Brain FA map derived from the region of interest analysis: (a) optic nerve, (b) optic radiation. We set the full width half maximum (FWHM) as 30.

**Figure 3:**
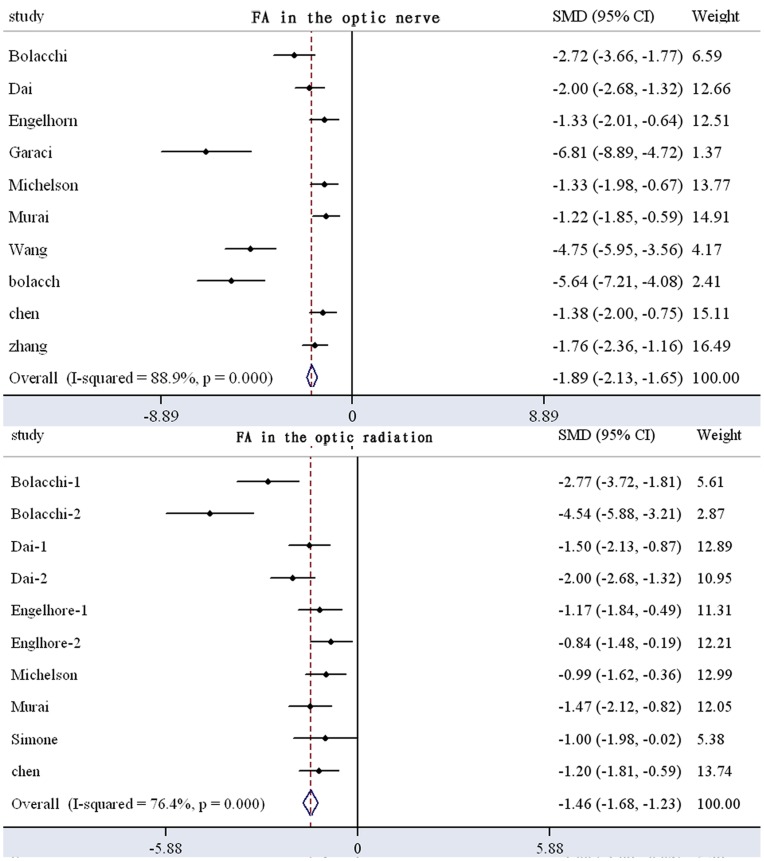
Meta-analysis results of FA in the optic nerve and optic radiation in patients with glaucoma compared with controls (data from Table 2). There is an apparent trend toward high heterogeneity (P<0.05, and the diamond shape meaning significant difference appears on the left side;I^2^>50%). FA: fractional anisotropy; SMD: standardized mean difference; CI: confidence interval.

**Figure 4:**
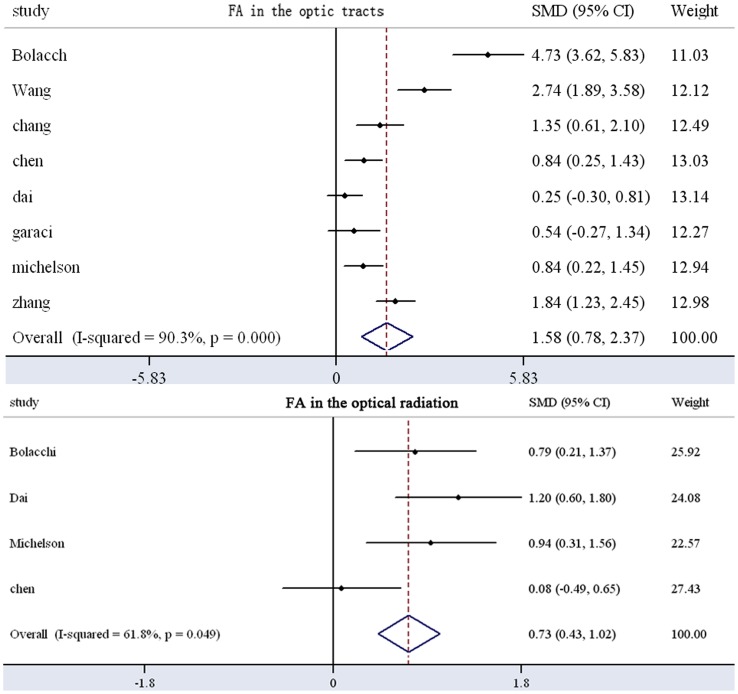
Meta-analysis results of MD in the optic tracts and optic radiation of patients with glaucoma compared with controls (data from Table 3). A significant difference between patients and controls was revealed, with high heterogeneity (P<0.05 and the diamond shape appears on the right side; I^2^>50%). MD: mean diffusivity; SMD: standardized mean difference; CI: confidence interval.

It has been demonstrated that there is a laterality of results in ROI studies. We extracted ROI-type studies and obtained FA and MD results in the optic nerve and optic radiation (Figure 2). Eleven studies with a total of 202 glaucoma patients and 218 controls were included in the meta-analysis of the FA in the optic nerves, which revealed a significant FA decrease in glaucoma patients (SMD: 1.89±0.34) with high heterogeneity (Figure 3). Nine studies involving 201 patients and 210 controls revealed a significant decrease in FA in the optic radiation of patients with glaucoma (SMD: 1.46±0.23) (Figure 3). Finally, eight studies with 192 patients and 167 controls revealed a significant increase in MD in the optic tracts of glaucoma patients (SMD: 1.58±0.80) (Figure 4). Five studies with 112 patients and 105 controls revealed a significant increase in MD in the optic radiation (SMD: 0.73±0.40) (Figure 4). These results indicate that FA and MD could be considered parameters of glaucoma severity.

### Subgroup Analysis

The FA in the optic nerve and optic radiation and the MD in the optic tracts and optic radiation showed a significant level of heterogeneity. To assess this heterogeneity, a fixed effects regression analysis was performed using patient characteristics (mean age, glaucoma stage) and acquisition details (field strength, b-value, acquisition matrix).

**Figure 5:**
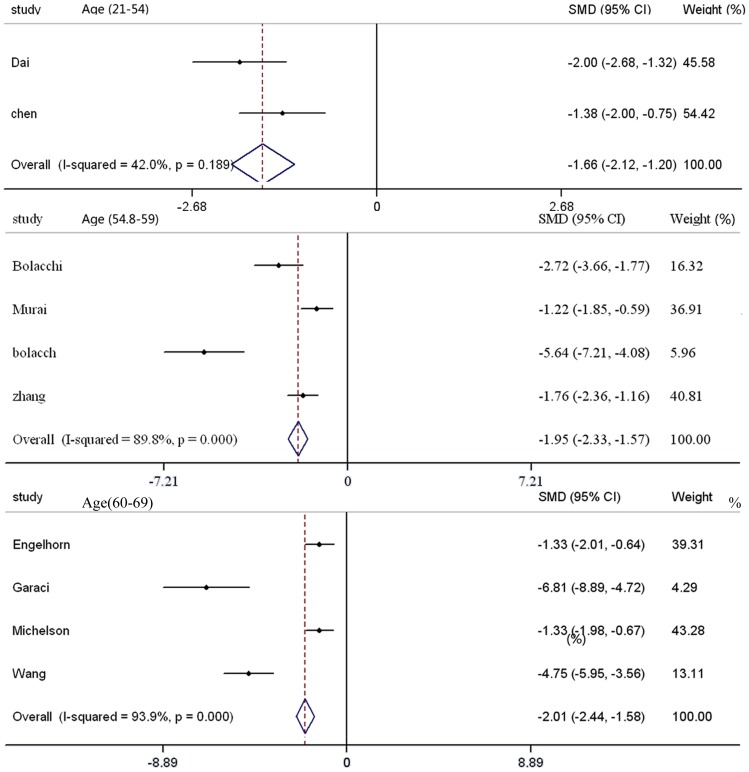
Results of an FA analysis with respect to age in the optic nerve of patients with glaucoma compared with controls. There is an apparent difference between three age groups (a: age 21–54 years, b: age 54.8–59 years, and c: mean age 60–69 years). We can conclude that there was deceasing FA of the optic nerve with increasing age in patients with glaucoma (P<0.05 and the diamond shape appears on the left side). FA: fractional anisotropy; SMD: standardized mean difference; CI: confidence interval.

**Figure 6:**
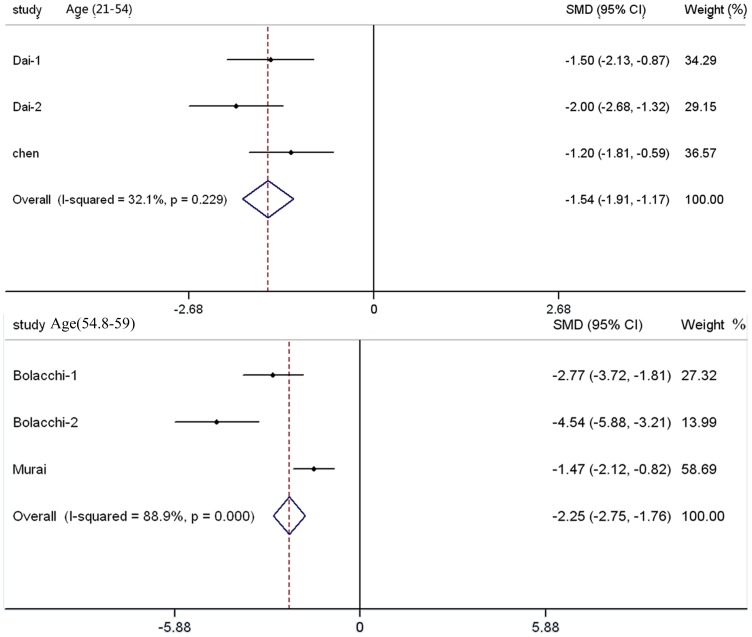
Results of an FA analysis with respect to age in optic radiation of patients with glaucoma compared with controls. There is an apparent difference between two age groups (a: age 21–54 years, b: age 54.8–59 years). We can conclude that there was decreasing FA of the optic radiation with increasing age in patients with glaucoma (P<0.05, and the diamond shape appears on the left side). FA: fractional anisotropy; SMD: standardized mean difference; CI: confidence interval.

**Figure 7:**
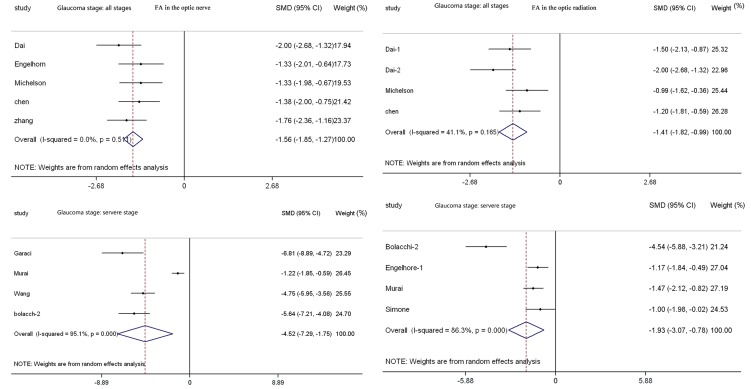
Results of an FA analysis with respect to glaucoma severity in the optic nerve (a) and optic radiation (b) of patients with glaucoma compared with controls. There is an apparent difference between two severity stage groups (a: all stages, b: severe stage). We can conclude that there was decreasing FA of the optic nerve with increasing glaucoma severity (P<0.05, and the diamond shape appears on the left side). FA: fractional anisotropy; SMD: standardized mean difference; CI: confidence interval.

Subgroup analysis was performed on the specified subgroups with comparable acquisition details. The analysis revealed a significant FA decrease in the optic nerve and optic radiation and an MD increase in the optic tracts and radiation. However, the results suggested weak or null relationships between the acquisition parameters and high heterogeneity (I2>50%). In contrast, the meta-regression revealed some evidence that the variability of the FA among studies was due to disease severity [10]. Regarding the comparison between controls and patients with glaucoma, FA decreased with increasing age and increasing glaucoma severity in the optic nerve and optic radiation (Figures 5, 6, 7(a) and 7(b)). The MD of the optic tracts and optic radiation in patients with glaucoma was increased compared with that in the control group and did not change according to disease severity.

## Discussion

Other DTI studies have revealed significant changes similar to those identified in this study, but this meta-analysis includes many more patients and is more powerful. Additionally, we aimed to reveal significant changes in DTI studies in patients with glaucoma. To the best of our knowledge, this is the first meta-analysis of DTI studies of glaucoma patients to demonstrate significant changes and provide evidence that DTI can detect white matter damage in patients with glaucoma.

In our meta-analysis, significantly reduced FA in the optic nerve and optic radiation and significantly increased MD in the optic tracts and optic radiation were found in the glaucoma group compared with the controls. Subgroup analyses further demonstrated that these findings were preserved in specified subgroups in studies assessing patients with different stages of glaucoma and mean ages. Regarding the different types of DTI studies, analyses of hypothesis-free studies revealed a progressive decrease in FA and an increase in MD with age in both ROI and whole-brain histograms [32], and ROI analysis was found to be useful for investigating specific white matter tracts or areas[33], [34]. Thus, in our meta-analysis, the selection of DTI studies with a priori-defined design analysis was rationalized, and our findings support our hypothesis that DTI may be a potential tool with which to detect microstructural damage in the optic nerve and optic radiation. Our results showed a greater decrease in FA in the rightcerebralhemisphere, suggesting that the FA decrease in the optic nerve and optic radiation exhibits laterality. This laterality is mainly attributed to the interpretation that the ROIs are located primarily in the right cerebralhemisphere, which was reported in previous studies [35]. The significant FA decrease suggests the potential diagnostic ability of DTI in glaucoma patients. Our findings suggest that previous studies were unable to demonstrate significant differences because of differences in glaucoma severity and age.

Generally, FA decreases with age, and a lower FA indicates more mature white matter bundles and fiber integrity [36]. Nevertheless, the mechanism of FA decrease and MD increase in patients with glaucoma remains unclear. Decreases in FA may be caused by a decrease in axial diffusivity (λ_∥_ = λ_1_) or an increase in radial diffusivity (λ_⊥_ = (λ_2_+λ_3_)/2) [37].Moreover, a statistically significant decrease in λ1 associated with an increase in λ2 and λ3 has been revealed in the distal segment [10]. Previous studies suggest that increases in axial diffusivity could be indicative of axonal damage or degeneration and that increases in radial diffusivity could represent a retinal nerve fiber layer thickness defect [38]. Thus, the reduction in FA observed in glaucoma patients may indicate axonal damage and defects in retinal nerve fiber layer thickness. Furthermore, previous studies also speculated that an MD increase and FA decrease accompanied by increases in the principal diffusivities in the optic nerve segment proximal to the optic nerve head occur in the early disease stages of glaucoma. In contrast, in the distal segment, the decrease in FA was accompanied by a decrease in the largest principal diffusivity, λ1, and an increase in the intermediate and smallest principal diffusivities, λ2 and λ3 [10].

Age is generally considered to be an important factor that may influence the results of a quantitative diagnosis in glaucoma studies. Our results showed that FA decreased as age increased, consistent with studies that reported the inverse relationship between FA and age. Previous reports revealed increasing atrophy of the optic radiation with increasing age in patients with glaucoma compared with age-matched controls [39], [40]. Furthermore, our subgroup analysis provides evidence that age and glaucoma stage affect the sectional heterogeneity in patients with glaucoma. However, in the subgroup analyses, other variables, including field of view, b-value, and Magnetic field intensity showed weak or null relationships and high heterogeneity. Although we employed a random effects model to compensate for heterogeneity, the different methodologies used in these studies may be criticized.

In addition to age, disease severity also plays an important role in the quantitative diagnosis of glaucoma. Our outcomes showed that FA decreased as glaucoma stage increased and indicated findings consistent with previous studies. Previous studies reported that the radial diffusivity (RD) and FA seen with glaucoma of the optic nerve increased and decreased, respectively, with time after glaucoma induction [12]. It has also been reported that FA is a sensitive biomarker of tissue micro-structural organization [41], [42]. Moreover, Williams et al. found widespread and complicated changes in cortical brain structure, and the degree of these changes correlated with disease severity [43]. In primary open-angle glaucoma, FA of the optic radiation is linked to indices of glaucoma severity [44].

DTI may provide important diagnostic clues in patients with glaucoma. DTI of optic nerve injuries can provide a noninvasive method to assess nerve fiber damage [45] and confirm the pathogenesis and clinical diagnosis of glaucoma [6], [46]. In conclusion, DTI in patients with glaucoma may help to provide objective evidence to aid in the diagnosis and management of glaucoma.

### Limitations

Our study has several methodological considerations and limitations that must be acknowledged. Due to the characteristics of a meta-analysis, we were able to perform statistical analysis only at the level of studies, with no means of confirming that the glaucoma participants in the included studies actually exhibited the findings presented. The considerable heterogeneity that we found between studies may be attributed to methodological issues. In addition, we cannot deny the probable existence of data that we could not find. Furthermore, although we demonstrated significant FA reductions and MD increases in glaucoma patients with a large effect size, the large total number of participants included in the analysis may have helped small differences reach significance. Moreover, the statistical method used divides two sets of estimated mean differences by the average standard deviation to produce an SMD. There are many confounding clinical and methodological factors that may affect the results. Thus, the findings should be interpreted with caution. With regard to the strength of the magnetic field, although heterogeneity analysis of studies that utilized a 3-T scanner preserved the findings, analysis of studies that utilized a 1.5-T scanner did not show significance [47].

### Conclusions

In conclusion, our findings clearly demonstrate a significant FA decrease and MD increase in the optic nerve and optic radiation of glaucoma patients. The results provide evidence indicating thatFA and MD could be used as parameters for the diagnosis of glaucoma and that FA may be correlated with glaucoma severity and age.

## Supporting Information

Figure S1 **PRISMA Flow Diagram.**
(DOC)

Checklist S1 **PRISMA Checklist.**
(DOC)
