# Supplementary material for: Alteration of Fractional Anisotropy and Mean Diffusivity in Glaucoma: Novel Results of a Meta-Analysis of Diffusion Tensor Imaging Studies
Source: PLoS One. 2014 May 14;9(5):e97445. doi: 10.1371/journal.pone.0097445 (PMC4020845; doi:10.1371/journal.pone.0097445)
Supplement: Figure S1 — PRISMA Flow Diagram. (DOC) [file pone.0097445.s001.doc]

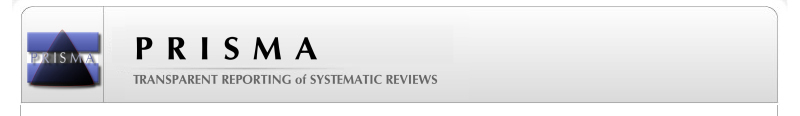
Flow Diagram

**Screening**

**Included**

**Eligibility**

**Identification**

Records identified through database searching
(n = 116)

Additional records identified through other sources
(n = 0 )

Records after duplicates removed
(n = 62 )

Records screened
(n =62)

Records excluded
(n = 0 )

Full-text articles assessed for eligibility
(n = 19)

Full-text articles excluded, with reasons*
(n = 51)

Studies included in qualitative snthesis
(n = 11 )

Studies included in quantitative synthesis (meta-analysis)
(n = 11 )

*Reasons:

- not human beings(n=10)

- with comorbidity or ill(n=6)

- not control group (n=1)

- Not DTI studies (n=5)

- conference articals (n=12)

- not original data (n=5)

- out of 2008 to 2013 (n=1)

- not related data or analysis (n=8)

- angle-closure glaucoma(n=1)

- whole brain analysis (n=2)
